# Moose Technique: A New Anatomical Perspective Treatment in Botulinum Toxin

**DOI:** 10.1111/jocd.70511

**Published:** 2025-11-04

**Authors:** Nabil Fakih‐Gomez, Cristina Muñoz‐Gonzalez

**Affiliations:** ^1^ Department of Surgery University of Salamanca Salamanca Spain; ^2^ Department of Facial Plastic and Cranio‐Maxillo‐Facial Surgery Fakih Hospital Khaizaran Lebanon

**Keywords:** botulinum toxin, dynamic wrinkles, incobotulinumtoxinA, injection technique, MOOSE technique, sad face

## Abstract

**Introduction:**

Botulinum toxin injections have transformed aesthetic treatments, improving patient confidence and emotional well‐being. Eyebrow position plays a vital role in facial expression, yet botulinum toxin use in the forehead can cause issues like eyebrow ptosis or asymmetry. Recent research emphasizes the importance of a comprehensive pre‐treatment assessment. The MOOSE technique, developed by the authors, incorporates new insights into muscle interactions to optimize botulinum toxin treatment for brow elevation while minimizing side effects like the omega sign.

**Methods:**

This study involved 202 patients (89.1% female, mean age 40) treated with the MOOSE technique, focusing on upper third facial wrinkles. Primary outcomes included brow position, forehead line severity, treatment duration, and patient satisfaction. Aesthetic outcomes were assessed using the Merz Aesthetic Scales (MAS) at baseline and at two weeks post‐treatment, corresponding to the peak efficacy of the toxin. Patient satisfaction was evaluated at both the two‐week and four‐month follow‐up.

**Results:**

The MOOSE technique resulted in a significant improvement in brow position, with an average gain of 2 points on the MAS. Patient satisfaction was remarkably high, with 100% of patients reporting a positive outcome at 15 days and 99% at four months. The average botulinum toxin dosage was 66 units per patient (range: 54–78 units). No cases of brow or eyelid ptosis were observed, and only one patient (0.49%) experienced transient edema. Interrater reliability of evaluations was excellent, with intraclass correlation coefficients ranging from 0.94 to 1.00 across all measured outcomes.

**Conclusion:**

The MOOSE technique provides an anatomically guided approach to botulinum toxin injections, enhancing aesthetic results while reducing complications. Its effectiveness underscores the value of individualized treatment based on muscle dynamics. Further research is needed to refine its application and assess long‐term effects.

## 1 Introduction

Aesthetic procedures such as botulinum toxin injections have transformed both cosmetic outcomes and patient well‐being by improving emotional appearance. Eyebrow shape and position are crucial elements to upper facial aesthetics and conveying emotions, gender traits, personality, and state of mind. Among facial features, the periocular region attracts the most attention, emphasizing the eyebrows' role in facial perception [1, 2, 3, 4, 5]. However, botulinum toxin treatments in the forehead can produce unpredictable effects, including brow asymmetry, ptosis, or a frozen look [6, 7].

Recent studies have highlighted the importance of comprehensive preassessment in botulinum toxin therapy, including evaluation of all core emotional expressions: anger, sadness, happiness, surprise, neutrality, and disgust [8]. The “omega sign,” a universal expression of sadness, is primarily influenced by the corrugator supercilii muscle (CSM) and frontalis, along with relaxation of the procerus, depressor supercilii (DSM), and medial orbicularis oculi (OOM) [9, 10, 11].

The CSM, traditionally viewed as the main brow depressor responsible for vertical glabellar lines [12, 13, 14, 15, 16, 17], has now been shown to also contribute to medial brow elevation through its interaction with the frontalis muscle and lateral brow depression through interactions with the OOM [11, 18, 19].

In a five‐year prospective study with 298 patients, the authors observed that targeted injections into the CSM and DSM improved brow elevation and expression lines, although omega‐shaped wrinkles often persisted [11]. Further research led to a classification of muscle control levels, enhancing aesthetic and emotional outcomes [8].

These findings call for a revision of current protocols, as many approaches still treat the CSM solely as a depressor. Effective botulinum toxin application requires a nuanced understanding of its functional anatomy. Technique also plays a key role: suboptimal targeting of neuromuscular plates may reduce treatment duration [20, 21, 22].

This paper presents the MOOSE technique, developed by author N.F.‐G., as a refined approach grounded in prior comparative and split‐face studies. By preserving the function of the central CSM and enhancing its synergy with the frontalis, the technique improves both medial and lateral brow elevation while reducing the omega sign [11]. The result is a more natural, balanced, and lasting aesthetic outcome.

## 2 Materials and Methods

Patients are eligible for this technique if they present with upper third facial wrinkles. The inclusion criteria include patients aged 25–65 years, without cognitive impairments or psychiatric conditions, and those who have undergone previous treatment with the traditional technique. This traditional approach involves treating the forehead along Cotofana's C‐line, leaving two lines of injection points 2 cm above the eyebrow, and addressing both the medial and lateral corrugator muscles, the procerus, and the lateral orbicularis oculi [23, 24, 25, 26]. Exclusion criteria include known allergies to botulinum toxin type A, human albumin, or sucrose, as well as generalized muscular diseases, infections, or inflammation at the injection site. Additional exclusions involve conditions affecting the stomatognathic system, such as trigeminal neuralgia, ptosis, or any pathological muscle activity reduction, as well as patients who have undergone facial surgery or aesthetic treatments with botulinum neurotoxin type A in the upper third of the face within the past 6 months.

**FIGURE 1:**
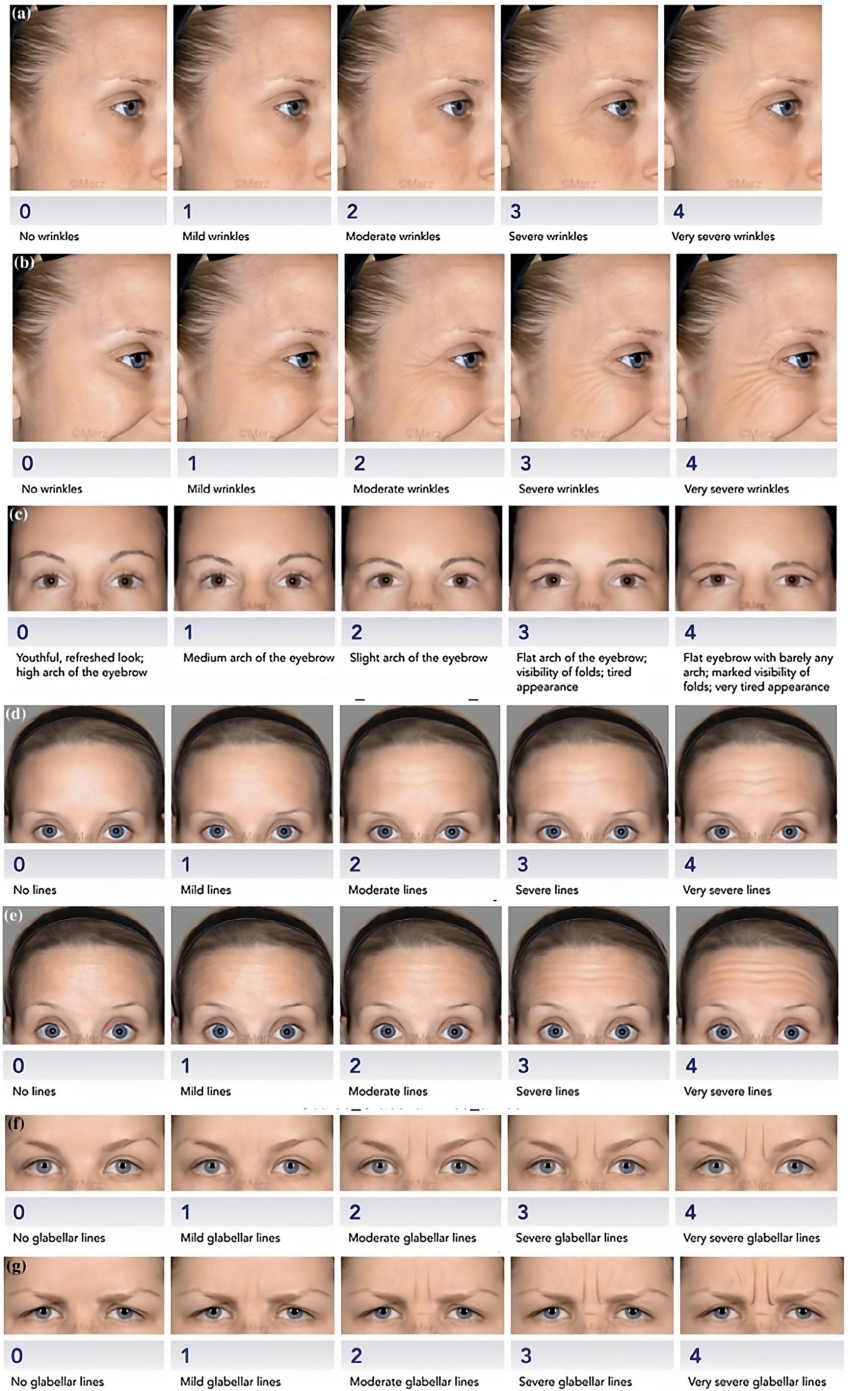
MERZ scale: (a) Crows' feet at rest scale, (b) Crows' feet dynamic scale, (c) Brow positioning scale, (d) Forehead lines at rest scale, (e) Forehead lines dynamic scale, (f) Glabella lines at rest scale, (g) Glabella lines dynamic scale [27].

Three primary outcomes were assessed using the MOOSE technique and evaluated with the Merz Aesthetic Scales (MAS) [27] (Figure 1), a validated instrument for measuring brow position and forehead line severity at rest and during movement [28, 29]. These outcomes included: aesthetic improvement, assessed at baseline and at two weeks, corresponding to the peak efficacy of the toxin; duration of the treatment effect, with a target of four months; and patient satisfaction, evaluated at both the two‐week and four‐month follow‐up in a cohort of 202 patients.

All assessments were conducted by one internal evaluator (C.M.‐G.) and one external evaluator (G.S.), both experienced in botulinum toxin treatments. The evaluation process involved a physical examination and standardized photographic documentation performed at baseline and at two weeks posttreatment. Patient satisfaction was assessed through a closed‐ended verbal question during the clinical evaluation: “Are you satisfied with the result obtained?” A positive response (“yes”) was recorded as indicative of satisfaction.

The initial assessment was conducted by the internal evaluator (C.M.‐G.), who reviewed the cases injected by the internal physician (N.F.‐G.). Following this, the external evaluator (G.S.) performed a secondary assessment of the cases.

For injections, a 100 U vial of incobotulinumtoxinA (Xeomin, Merz Pharmaceuticals GmbH, Frankfurt, Germany) was reconstituted with 2.6 mL of 0.9% sterile, non‐preserved saline to ensure that the final volume effectively delivered to the patient corresponded to 2.5 mL, accounting for the approximate 0.1 mL loss that typically remains within the dead space of the needle and syringe during preparation. Precise injection technique is essential, with the total number of units and volume customized for each patient according to the dosing scheme outlined in the following section.

This approach involves several steps: evaluating each patient's facial anatomy and dynamic function, marking accurate injection points, determining the appropriate dosage, and aligning the treatment with the patient's aesthetic goals. Patients' preferences should be considered, as eyebrow shape and position play significant roles in conveying femininity and masculinity [5]. Using a mirror, patients are guided to observe the natural shape of their eyebrows, while the practitioner points out any asymmetries or imbalances. By asking the patient to relax and then contract the frontalis muscle, the effect of paralysis can be simulated through varying pressure at the marked points, enabling the patient to visualize the potential outcomes in the mirror. The natural brow shape should be maintained both at rest and during maximum contraction, allowing the patient to make an informed decision about the desired outcome.

A comprehensive assessment is critical to achieving optimal aesthetic results, considering individual anatomy, muscle function, habitual facial movements, and a wide range of expressions. Muscle mass and strength should also be considered [20]. Forehead evaluation must include observations of muscle behavior both at rest and during maximum contraction, as well as during facial expressions like surprise, anger, happiness, disgust, and sadness [8]. Additionally, it is important to mark the eyebrow position, assess maximum height, and evaluate for excess skin on the upper and lower eyelids [30].

IncobotulinumtoxinA dosing and injection site distribution should reflect variations in muscle pattern, size, mass, and movement to ensure optimal outcomes.

**FIGURE 2:**
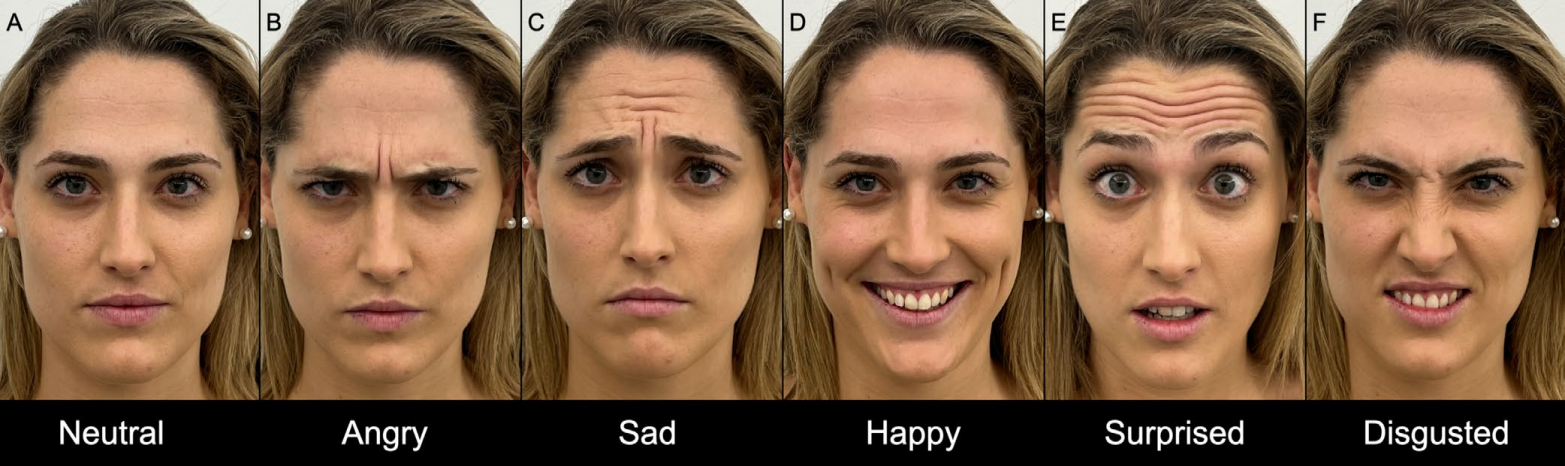
Illustration of the recommended pre‐treatment assessments with photographic documentation, featuring frontal view photographs that capture a full range of facial expressions in a 26‐year‐old female subject: (A) Neutral, (B) Angry, (C) Sad, (D) Happy, (E) Surprised, and (F) Disgusted [8].

Photographic documentation is taken before any aesthetic treatment and should be reviewed with the patient during the pretreatment assessment (Figure 2). Visual scales are useful in setting realistic expectations and facilitate discussions by providing objective references to facial landmarks, signs of aging, and any existing asymmetry [31].

For the assessment, both the patient and the assessor should sit face‐to‐face at eye level. Observing functional anatomy during maximum muscle contraction is important, particularly in identifying static lines at rest, asymmetries, or signs of eyebrow and/or eyelid ptosis [30]. It is essential to determine if the patient exhibits the omega sign while frowning, as this will guide the targeting of these lines. Although anyone can display the omega sign, we adhere to the classification established by the authors, which categorizes it as a weak, controllable, or automatic gesture [8].

### 2.1 Moose Technique Markings

**FIGURE 3:**
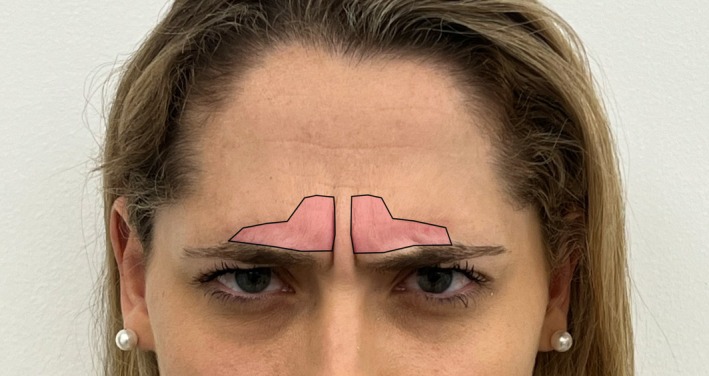
Initial marking of the corrugator muscles, represented in light pink, is performed while the patient frowns, allowing for precise identification of their location and dynamic activity.

**FIGURE 4:**
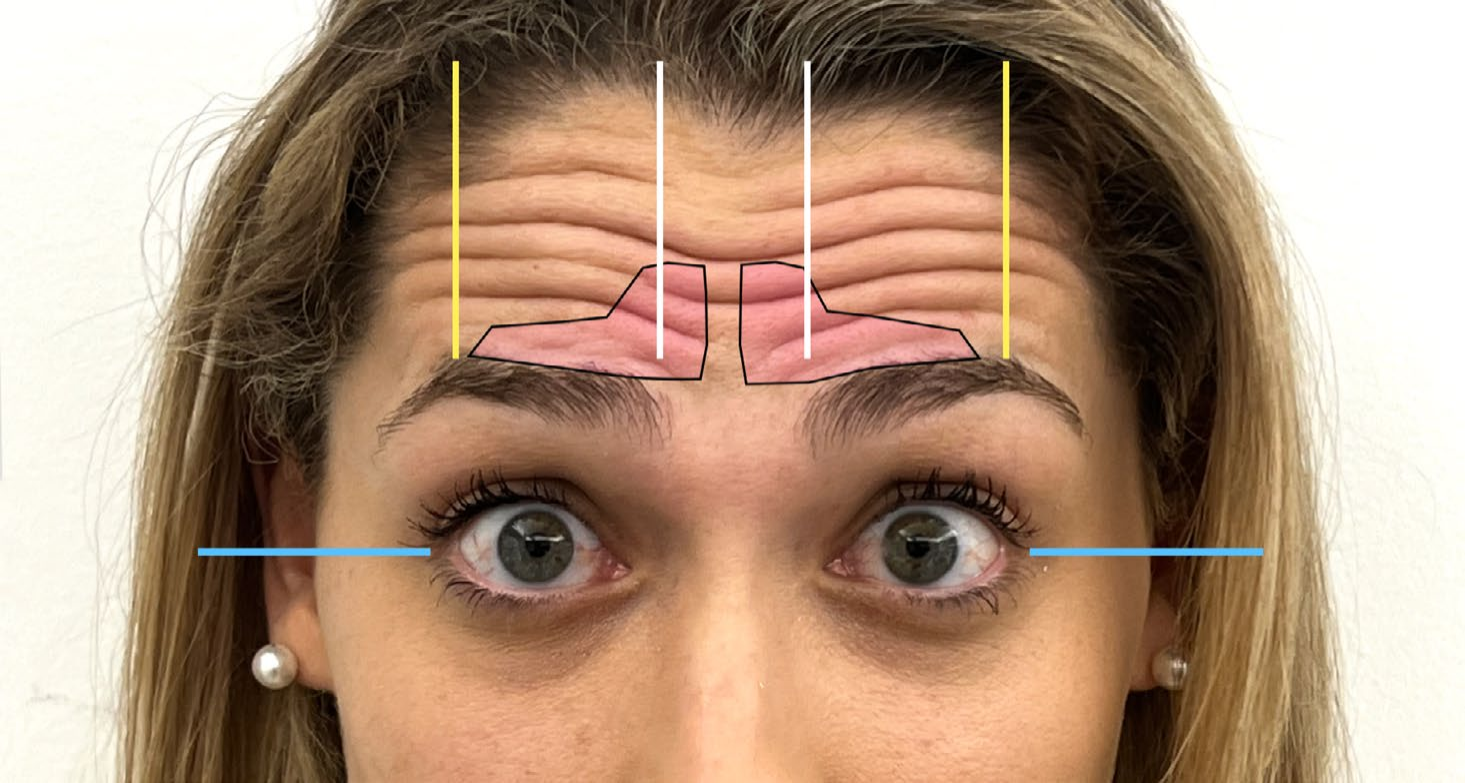
With the patient raising their eyebrows, blue lines indicate the lateral canthal trajectory, white lines trace the vertical alignment from the brow head to the hairline, and yellow marks identify the highest point of the brow arch.

We begin by marking the corrugators, asking the patient to frown (Figure 3). Next, the patient raises their eyebrows, and we mark two lines following the highest peak of the brow, with two additional lines extending from the heads of the medial brows toward the hairline, and two more lines along the lateral canthal line (Figure 4).

**FIGURE 5:**
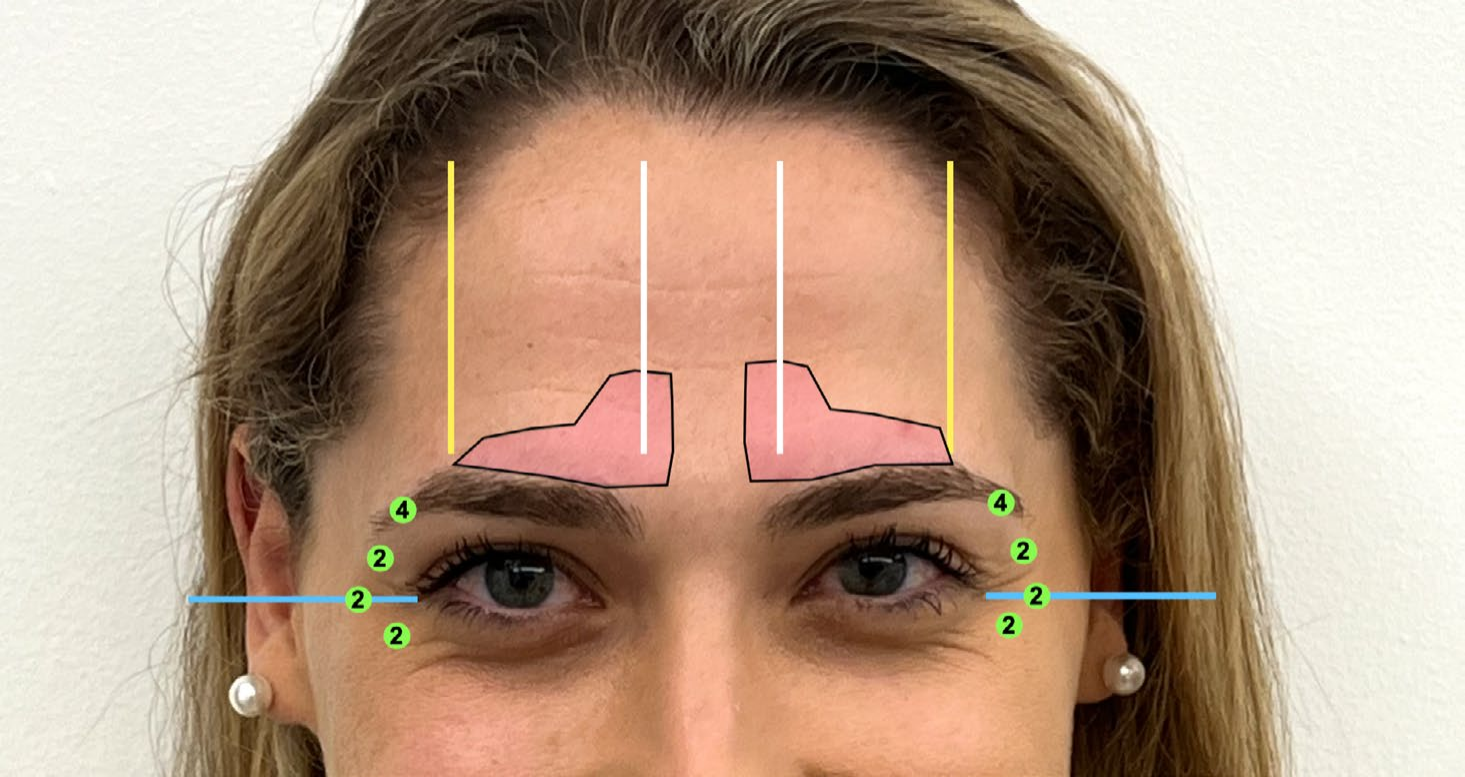
Injection points for crow's feet are marked with the patient smiling, including a lateral brow hairline point and three L‐shaped points along the OOM. All injection points are marked in green, indicating a superficial depth, and the number of incobotulinumtoxinA units used is represented inside each circle.

To address crow's feet, the patient is asked to smile, and four classic L‐shaped points are marked to target the OOM. One point is placed at the hairline level of the lateral brow, a second on the extended canthal line, a third midway between these two, and a fourth, more medial and inferior, completing the characteristic ‘L’ shape (Figure 5).

**FIGURE 6:**
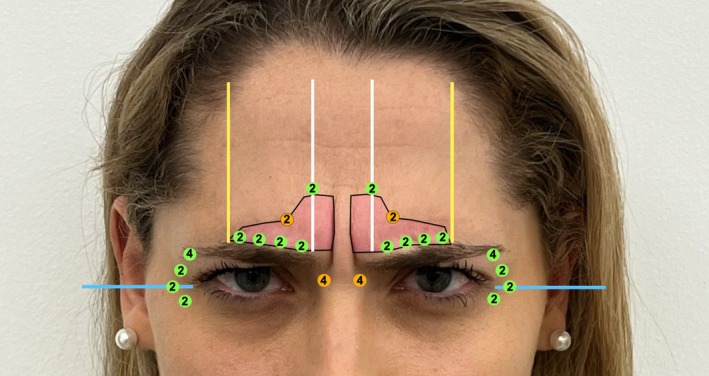
Injection point markings for the DSM, corrugator (both low and high lateral portions), and the central lower frontalis during active frowning. Injection points are color‐coded by depth: Green indicates superficial injections, orange denotes medial‐depth injections. The number of incobotulinumtoxinA units administered is shown within each circle. Injections in the lower corrugator should be performed very superficially, inserting the needle no more than 1 mm, and typically administering 1 to 2 units per point. The same caution applies to the DSM, where 2 to 4 units may be used depending on muscle strength and anatomical variation.

The DSM is assessed, and two points are placed while the patient frowns. The procerus is not marked. The horizontal portion of the corrugator (low lateral corrugator) is marked with 2–4 points, depending on its length, following the vertical contraction lines that appear when frowning. The high oblique portion (high lateral corrugator) is marked with one point on each side. Two points are placed on the central lower frontalis muscle, following the central white lines, marking where the corrugator ends during a frown, which aligns with these lines (Figure 6).

**FIGURE 7:**
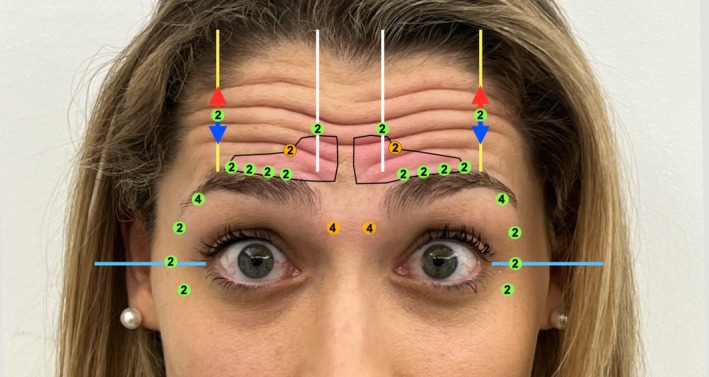
Identification of the brow's highest peak during a “surprise” expression, with injection points aligned to address asymmetry. Red and blue arrows illustrate potential vertical movement. Injection depths are color‐coded, and incobotulinumtoxinA units are indicated within each circle.

The next step is to identify the highest peak of the brow during a “surprise” expression. The desired eyebrow height is determined here, noting that injections closer to the hairline allow for more mobility but may leave wrinkles depending on the patient's muscle contraction pattern. Conversely, injections closer to the brow hairline provide more aggressive blockage. It is important to ensure both injection points are at the same height or adjusted if there is pre‐existing asymmetry (Figure 7).

**FIGURE 8:**
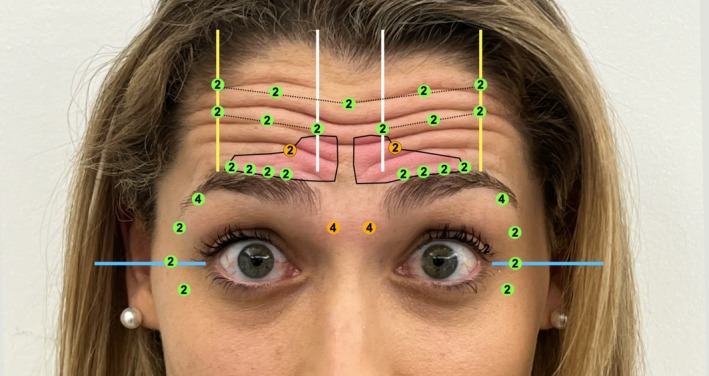
Once the maximum brow peak points are established and considering the low frontal points that align with the highest border point of the CSM, we will mark a midpoint between these two on each side. Superiorly, two additional points will be placed near the hairline, approximately 1 cm above the high brow peak points. A medial frontal point will also be marked, with equidistant points placed between this and the previous ones. For patients with a larger forehead, an additional line of points may be added.

Once the maximum brow peak points are established and taking into account the low frontal points that align with the contraction points of the corrugator muscle, we will mark a midpoint between these two on each side. Superiorly, two additional points will be placed near the hairline, approximately 1 cm above the high brow peak points. A medial frontal point will also be marked, with equidistant points placed between this and the previous ones (Figure 8). For patients with a larger forehead, an additional line of points may be added.

Each injection point is selected as part of a fully customized treatment plan, with dosage adjusted according to muscle strength and the desired degree of relaxation. Stronger muscles such as the lateral brow point and the DSM typically require 4 units. Moderately active muscles generally respond well to 2 units, which are applied to the remaining points. Injections into the lower portion of the corrugator should be performed with extreme precision, inserting the needle no more than 1 mm deep and delivering 1–2 units per point. In areas where muscle activity is minimal or absent, no botulinum toxin is administered, ensuring targeted, balanced, and effective results.

### 2.2 Statistical Analysis

The data assessed are of a prospective nature. Therefore, the patients' central frown lines, frontal lines, crow's feet, and brow position were evaluated at baseline and again at two weeks during physical assessments using the MAS for classification. Each evaluation was conducted twice: once by the internal evaluator physician and once by an independent external physician. To reflect both the degree of correlation and agreement between the ratings, the intraclass correlation coefficient (ICC) was employed. In this study, the ICC was calculated using a two‐way random effects model.

## 3 Results

From January 6, 2022, to January 6, 2024, a total of 202 patients underwent the MOOSE technique, administered to the upper third of the face. The study population consisted of 180 female patients (89.1%) and 22 male participants (10.9%), with a mean age of 40 years (range: 25–65 years). Eight cases (3.9%) had well‐controlled hypothyroidism, while seven cases (3.5%) had beta thalassemia minor. Additionally, five patients (2.4%) had a prior medical history of well‐controlled Type II diabetes, three patients (1.5%) had hypercholesterolemia, and seven patients (3.5%) had well‐controlled hypertension.

The MOOSE technique was administered as previously defined in all 202 cases (100%). The average dose of botulinum toxin type A (BONT‐A) injected was 66 units per patient (range: 54–78 units). Adverse events were monitored throughout the study period, and standardized follow‐up photographs were obtained 15 days post‐injection. Treatment outcomes were independently evaluated using the MAS by both an internal evaluator and an external practitioner, revealing an average improvement of 2 points at the 15‐day follow‐up. However, further improvement was limited in some patients, particularly older individuals with established static lines, where the maximal effect of the toxin was insufficient to completely smooth deep‐set wrinkles, thereby capping the achievable MAS score.

**FIGURE 9:**
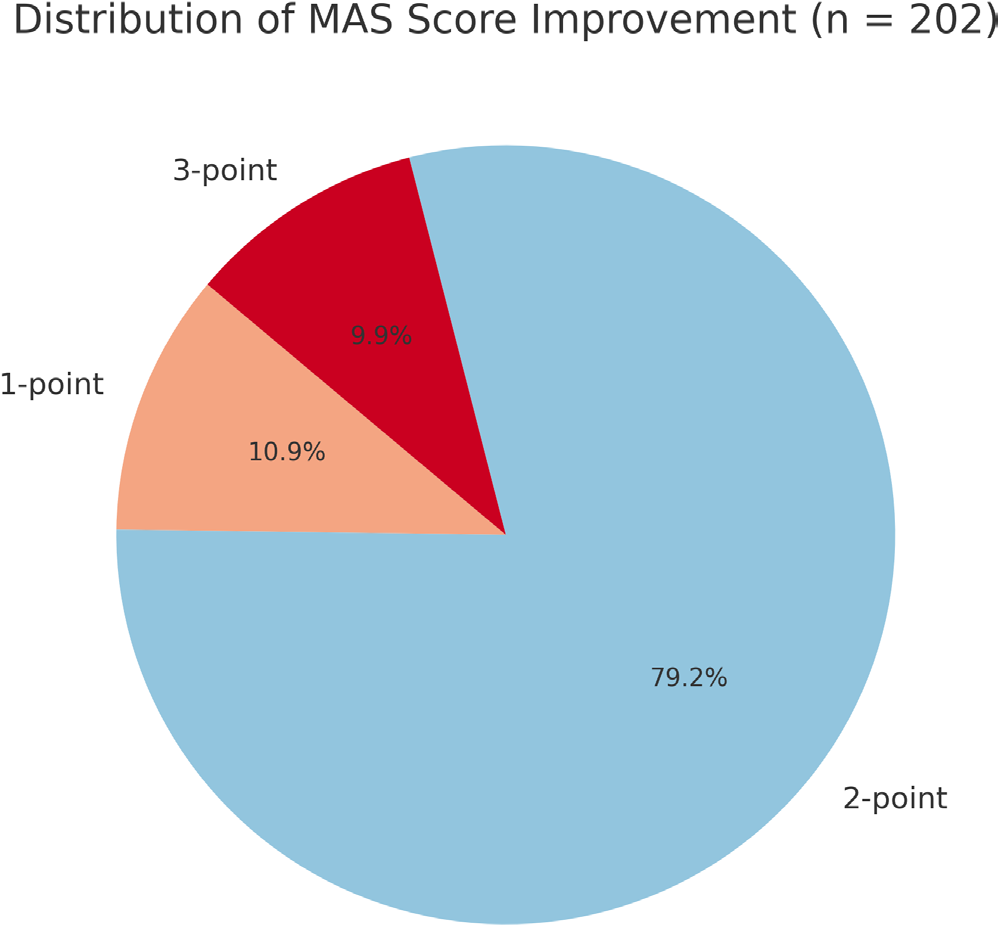
Distribution of MAS score improvement among patients (*n* = 202). A total of 160 patients (79.2%) achieved a 2‐point improvement, 22 patients (10.9%) improved by 1 point, and 20 patients (9.9%) improved by 3 points, illustrating a predominantly high clinical response to treatment.

To further characterize the clinical response, authors conducted a responder analysis revealing that 160 patients (79.2%) achieved a 2‐point improvement, 22 patients (10.9%) improved by 1 point, and 20 patients (9.9%) demonstrated a 3‐point improvement. Notably, all patients experienced at least a 1‐point improvement, underscoring the robustness and consistency of the treatment effect (Figure 9).

The cohort was predominantly female, with 180 out of 202 patients (89.1%) being women. The mean MAS score improvement was 1.98 points in female patients (standard deviation: 0.45) and 2.06 points in male patients (standard deviation: 0.50), with no appreciable gender‐related disparity in treatment efficacy.

**FIGURE 10:**
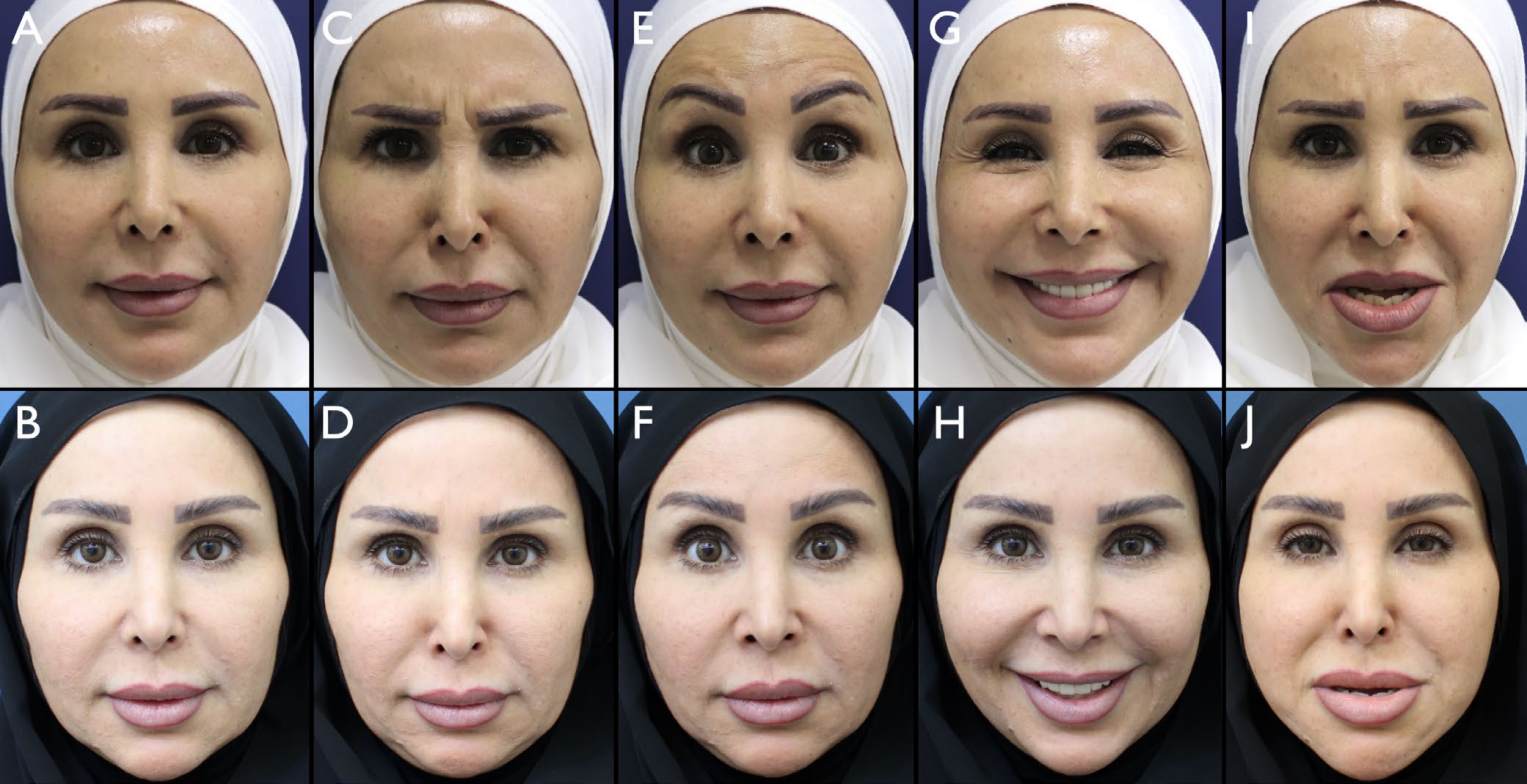
Female patient, 45 years old. Panels A, C, E, G, and I show the patient's facial expressions in the following sequence: Neutral, frowning (anger), eyebrow elevation (surprise), smiling (happiness), and a simulated sad expression. Panels B, D, F, H, and J depict the corresponding expressions 15 days after treatment.

**FIGURE 11:**
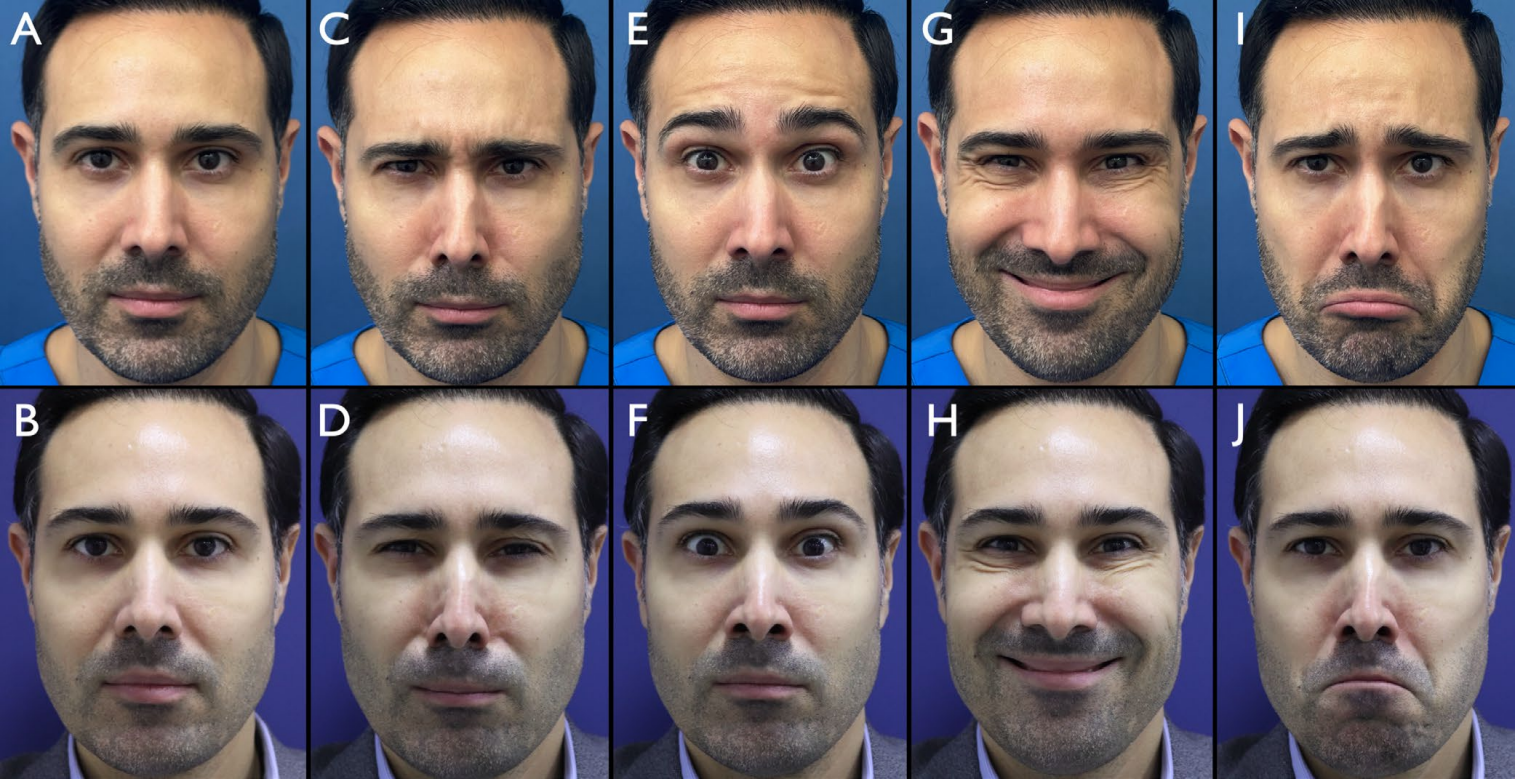
Male patient, 43 years old. Panels A, C, E, G, and I show the patient's facial expressions in the following sequence: Neutral, frowning (anger), eyebrow elevation (surprise), smiling (happiness), and a simulated sad expression. Panels B, D, F, H, and J depict the corresponding expressions 15 days after treatment.

**FIGURE 12:**
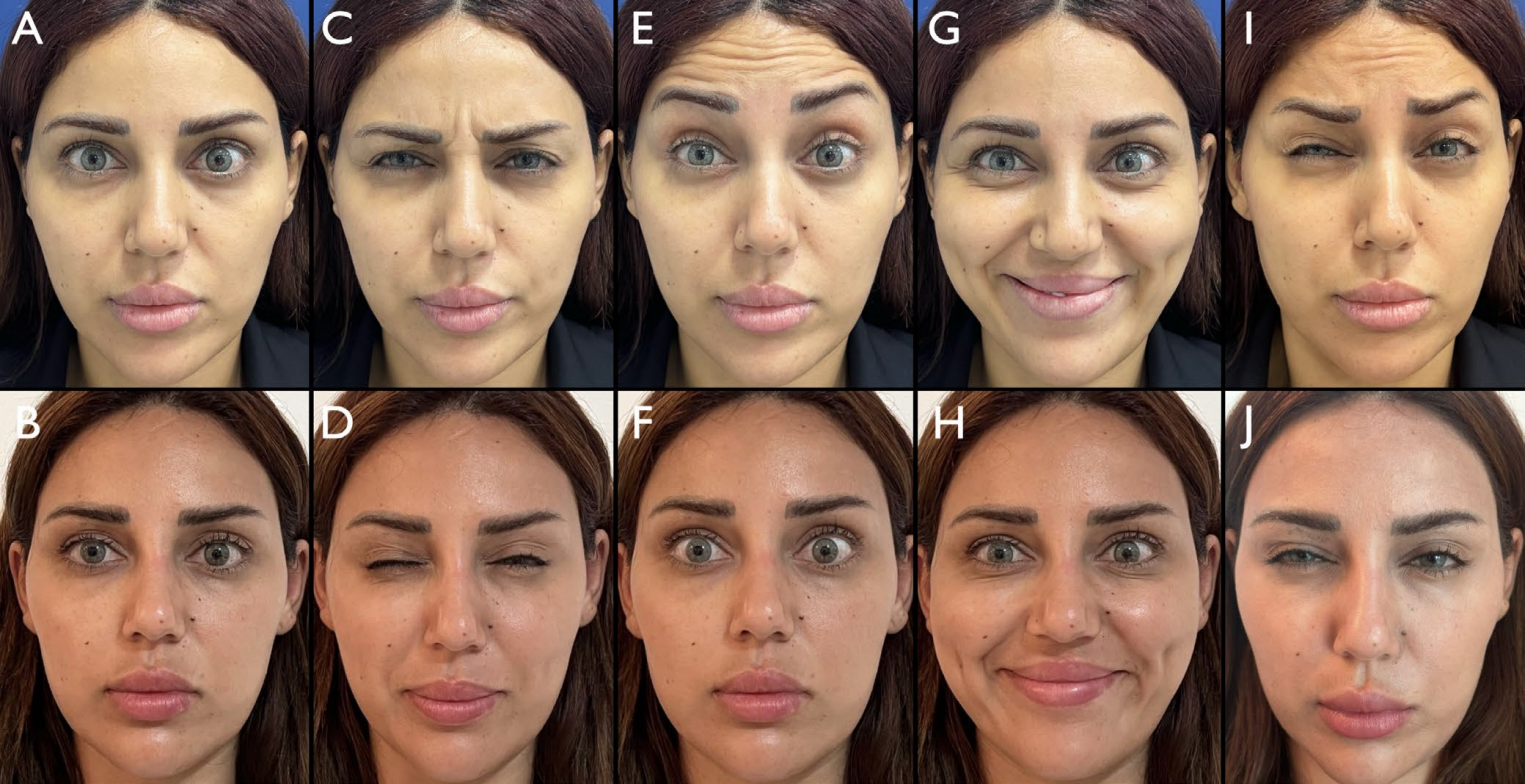
Patient female 33 years old. Panels A, C, E, G, and I show the patient's facial expressions in the following sequence: Neutral, frowning (anger), eyebrow elevation (surprise), smiling (happiness), and a simulated sad expression. Panels B, D, F, H, and J depict the corresponding expressions 15 days after treatment.

Overall, 180 patients (89.1%) reached or exceeded the 2‐point improvement threshold. The overall standard deviation across the full cohort was 0.46, and the 95% confidence interval for the mean MAS improvement was [1.93–2.05], indicating a narrow and reproducible range of response. These statistical findings reinforce the reliability and clinical utility of the MOOSE technique across genders (Figures 10, 11, 12).

Patient satisfaction was notably high, with 100% of patients reporting a positive outcome at the 15‐day follow‐up and 99% at the four‐month follow‐up, where only two patients responded negatively to the closed‐ended question: “Are you satisfied with the result obtained?” These subjective satisfaction reports were consistent with the objective improvements observed on the MAS. Importantly, no cases of brow or eyelid ptosis were observed with the use of incobotulinumtoxinA. One patient (0.49%) experienced transient edema in the treated area during the first two weeks, which resolved spontaneously without intervention and did not recur. No other adverse events were reported.

### 3.1 Interrater Reliability of the CR‐MAS Assessments

The patients' central frown lines, forehead lines, crow's feet, and brow position were evaluated at baseline and again two weeks later during physical assessments, utilizing the MAS for classification [27]. To determine the consistency between the evaluations recorded by the internal evaluator and those from an independent physician, an interrater reliability analysis was conducted using the intraclass correlation coefficient (ICC). This analysis was performed using a two‐way random effects model [32, 33]. The agreement between the two raters was outstanding, with ICC values at baseline and two weeks post‐treatment for improvements in central frown lines, forehead lines, crow's feet, and brow position recorded at 0.99, 0.94, 1.00, and 0.98, respectively.

## 4 Discussion

Although traditionally regarded as the primary depressor of the brow and responsible for vertical glabellar lines, recent evidence highlights a more nuanced function of the CSM. Its complex interaction with adjacent muscles suggests a dual role in both medial brow elevation and lateral brow depression, depending on the muscular context. These findings, supported by a large prospective study and a functional classification system, underscore the importance of individualized assessment when planning botulinum toxin treatments [8, 11, 12, 13, 14, 15, 16, 17, 18, 19].

**FIGURE 13:**
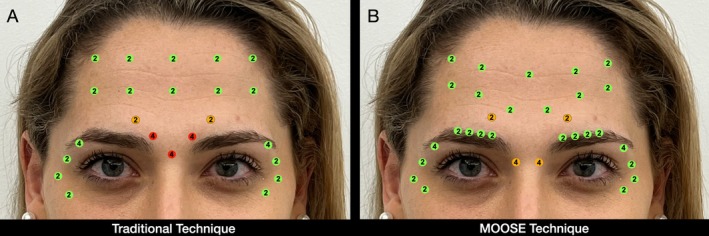
Comparative injection markings of the traditional and MOOSE techniques in the same patient at rest: (A) Traditional technique with frontal points following the 2 cm C‐line, targeting the middle corrugator and procerus muscles; (B) MOOSE technique, which omits injections in the procerus and middle corrugator, incorporates low lateral corrugator and DSM points, and positions frontal injections slightly lower. Injection points are color‐coded by depth: Green for superficial, orange for medial, and red for deep, with Xeomin units indicated inside each circle.

The MOOSE technique builds on this updated understanding by introducing three key innovations compared to traditional approaches (Figure 13). First, it redefines the role of the CSM, recognizing its primary function as a medializer rather than a depressor of the brow. While the CSM contributes to lateral brow depression when combined with the lateral OOM, its interaction with the frontalis muscle elevates the medial brow region [11]. Therefore, the authors strongly discourage blocking the medial CSM, as this may result in an exaggerated drop of the central brow.

Second, the authors found that targeting the DSM facilitates a natural brow lift by allowing the frontalis muscle to elevate the brow unimpeded, making this point fundamental to the treatment. During this process, the diffusion of botulinum toxin to the procerus muscle occurs naturally, eliminating the need for additional injections in that area [10, 34]. This leads to the final key distinction point of the MOOSE technique: the decision not to treat the procerus muscle directly. This conclusion is further supported by findings indicating that the neuromotor plates of the procerus are often located laterally, near the DSM [22].

As observed in a previous study, targeting the middle corrugator with traditional techniques often results in excessive weakening of the central brow area, leading to unintended medial brow descent. This imbalance may provoke the so‐called “Mephisto” effect, characterized by over‐elevation of the lateral brow. To counteract this, practitioners may lower the lateral brow peak, ultimately causing both the medial and lateral brow segments to descend, compromising aesthetic harmony [23, 25].

In contrast, the authors' technique avoids complete paralysis of the CSM, allowing the medial brow to remain elevated and contributing to a more balanced and natural brow lift. This approach also permits injections along a lower frontal line, without strict adherence to the traditional C‐line [26]. By preserving medial CSM activity and selectively targeting the DSM, a controlled interplay between elevating and depressing forces is achieved, minimizing the risk of brow ptosis and the Mephisto effect (Figure 13).

In a previous study involving 298 patients, the authors tested various injection approaches before refining and developing the MOOSE technique [11]. The pivotal realization that the CSM should not be classified as a brow depressor led to a paradigm shift in botulinum toxin injection strategy. This innovation addressed complications such as the formation of omega lines, which were managed by selectively blocking the lower frontalis muscle (above the high portion of the CSM), thereby reducing central horizontal forehead lines (omega wrinkles).

By recognizing the CSM's role as a medializer rather than a depressor, this technique offers a more anatomical approach, resulting in improved aesthetic outcomes and fewer complications such as brow ptosis and omega‐shaped wrinkles.

Since incobotulinumtoxinA (Xeomin) exhibits limited diffusion [35, 36, 37, 38], it allows for safe injections below the mid‐forehead line without a significant risk of brow ptosis. However, as diffusion properties vary between botulinum toxin formulations, caution is advised when applying this technique with other products. Toxins with broader diffusion profiles may increase the risk of undesired effects, such as brow or eyelid ptosis. Therefore, further studies are needed to validate the safety and efficacy of this approach when using alternative formulations, as well as to assess its long‐term impact on facial dynamics.

In this study involving 202 patients treated with the MOOSE technique, the demographic analysis revealed a predominance of female participants (89.1%) with a mean age of 40 years. The majority of patients were generally healthy, with only a small percentage presenting with pre‐existing conditions such as well‐controlled hypothyroidism, beta thalassemia minor, and Type II diabetes. This highlights the safety of the procedure in a clinically stable population.

The MOOSE technique was administered consistently across all cases, resulting in an average improvement of 2 points on the MAS [27] 15 days post‐treatment, suggesting a meaningful enhancement in aesthetic outcomes. Furthermore, 100% of patients reported satisfaction at the two‐week follow‐up, and 99% at four months, highlighting the technique's high level of acceptance and sustained perceived effectiveness over time. The low incidence of adverse events, with only one case (0.49%) of transient edema that resolved spontaneously, underscores the technique's safety profile.

These findings support the MOOSE technique as a valuable option in aesthetic medicine, demonstrating not only its efficacy but also its safety in a diverse patient population. The positive patient feedback and minimal adverse effects suggest that this approach could be integrated into standard practice for enhancing aesthetics. Future studies with larger sample sizes may provide further insights into the long‐term effects and potential benefits.

**FIGURE 14:**
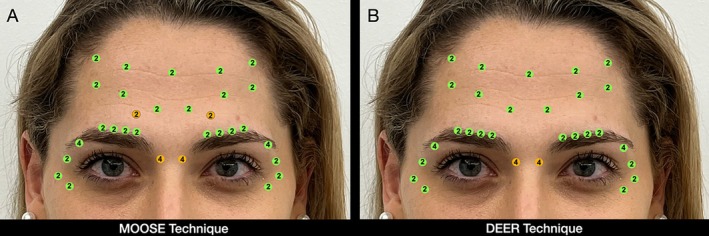
Comparative injection points between the MOOSE and DEER techniques: (A) MOOSE technique, and (B) DEER technique, applied in patients with a weak sad face pattern, in which the HLC points are omitted.

Note that in patients with a weak sad face and not being automatic nor controlled sad face patients, it is possible to perform the same technique without adding the high lateral corrugator (HLC), naming this technique the DEER technique (Figure 14). As previously mentioned, this option is reserved for selected patients, since omitting the HLC point in individuals with strong frontalis and medial corrugator activity may unmask or accentuate a “sad face” or omega sign during spontaneous frowning [8]. This occurs because the untreated medial and lateral corrugator, acting in synergy with a strong frontalis, pulls the medial brow upwards in a paradoxical hyperkinetic pattern, leading to an exaggerated central elevation that mimics or enhances the appearance of sadness or fatigue.

**FIGURE 15:**
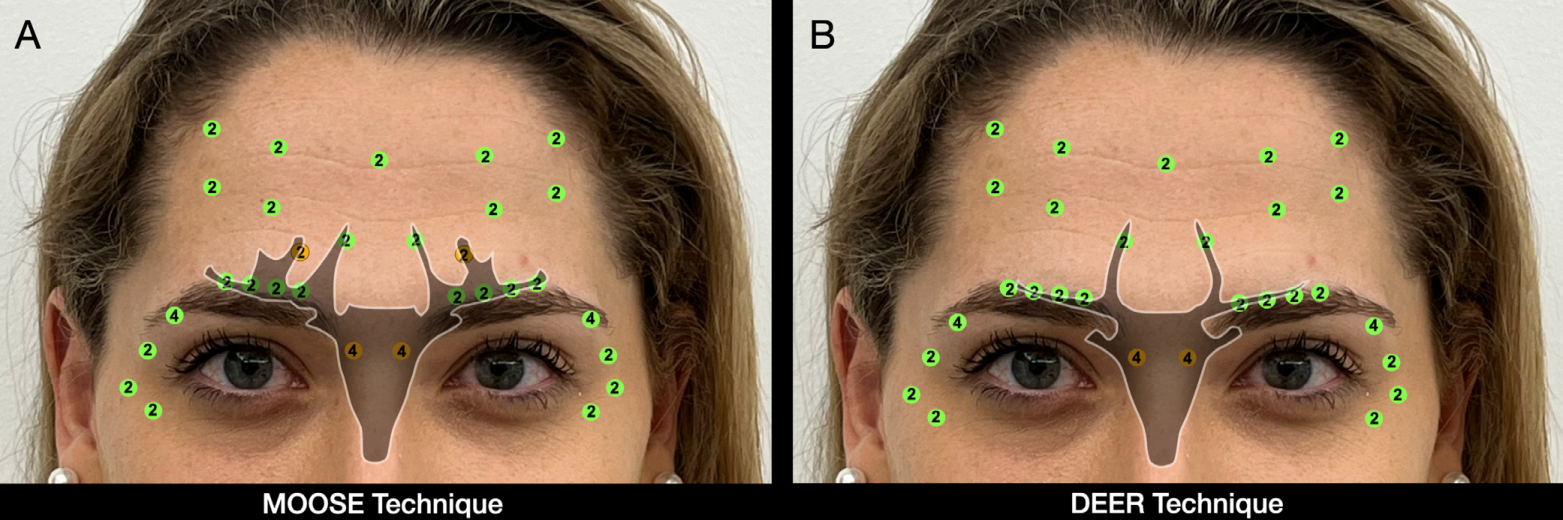
Comparison of central injection markings resembling animal horns, broader and more prominent in the MOOSE technique (A) and slimmer in the DEER technique (B). This visual analogy highlights the main difference between both approaches.

The names MOOSE and DEER are derived from the resemblance to the horns of animals at the central points in the glabellar region, which is the main difference between the two techniques (Figure 15).

As we learn more about facial anatomy, it's important to update our techniques in aesthetic medicine, and the MOOSE technique offers a meaningful improvement. The authors are currently investigating the role of the parietotemporal muscle as a potential anatomical retainer of frontalis tension, possibly contributing to the downward pull of the galea during contraction, an effect previously described as the “accordion phenomenon.” This mechanism may help explain persistent forehead lines in some patients and support more effective brow elevation strategies [39]. In 2020, Cotofana et al. introduced the concept of a “line of convergence,” proposing bidirectional frontalis contraction with opposing movements in the upper and lower forehead [5]. While this model generated important discussion, it departs from classical muscle mechanics. More recent evidence by Rahman et al. [40], based on AI‐driven simulations and anatomical analysis, supports a unidirectional contraction model, attributing skin movement to elastic resistance rather than opposing forces. These findings underscore the importance of anchoring new clinical interpretations in validated anatomical research [40]. In parallel, the authors are exploring the effects of hyperdilution of botulinum toxin combined with more distributed injection points [41]. Previous studies have shown that increasing the number of injection sites impacts more neuromotor plates, thereby prolonging the effect of the toxin [42, 43, 44]. This concept is also grounded in research highlighting the importance of targeting the correct muscle with precise technique to maximize the duration of the treatment [20, 21].

This study has some limitations that should be acknowledged. Patient satisfaction was assessed using a single closed‐ended verbal question, which, although standardized and easy to implement in clinical practice, does not capture the full range of patient‐reported experiences. The absence of a control group treated concurrently with the traditional technique limits direct comparative analysis. The study population was predominantly female and within a limited age range, which may not fully reflect the diversity of patients undergoing upper third facial botulinum toxin treatments. Lastly, although assessments were conducted by independent evaluators, the authors' involvement in the development and application of the technique could introduce an element of bias, despite efforts to ensure objectivity.

## 5 Conclusion

This study presents the MOOSE technique as an optimized approach to botulinum toxin application, enhancing eyebrow elevation while minimizing adverse effects like the omega sign. In 202 patients treated over two years, the technique showed significant aesthetic improvement, high satisfaction, and minimal complications, supporting its integration into standard aesthetic practice.

## Disclosure

Data Policy: For this type of study, we don't have data to deposit in a public repository.

## Ethics Statement

The Ethics Committee of Fakih Hospital reviewed and approved the protocol for this prospective study, assigning reference number 00001145 on December 18, 2021. All treatments were performed in adherence with the Declaration of Helsinki and in accordance with the standards of good clinical care following local guidelines and regulations. This article does not contain any studies with animals performed by any of the authors.

## Consent

All patients included in this study provided written informed consent for accessing their patient charts and extracting their data for the purposes of this study. No charts were accessed if patients declined their participation in this study. All participants have provided consent for the publication of their photographs.

## Conflicts of Interest

The authors N.F.‐G. and C.M.‐G. are consultants for Merz Aesthetics (Frankfurt, Germany). N.F.‐G. and C.M.‐G. are joint first authors.

## Data Availability

The data that support the findings of this study are available from the corresponding author upon reasonable request.
